# Polymorphisms of the SERPINA1 gene are associated with higher mortality in a Brazilian cohort of ANCA-associated vasculitis patients

**DOI:** 10.1016/j.clinsp.2024.100524

**Published:** 2024-10-30

**Authors:** Henrique Ayres Mayrink Giardini, Valeria de Falco Caparbo, Isac de Castro, Andréia Padilha Toledo, Carmen Silvia Valente Barbas, Samuel Katsuyuki Shinjo, Rosa Maria Rodrigues Pereira

**Affiliations:** a Rheumatology Division, Hospital das Clínicas, Faculdade de Medicina, Universidade de São Paulo (HCFMUSP), São Paulo, SP, Brazil; b Pneumology Division, Hospital das Clínicas, Faculdade de Medicina, Universidade de São Paulo (HCFMUSP), São Paulo, SP, Brazil

**Keywords:** Gene, Polymorphism, Mortality, Systemic vasculitis

## Abstract

**Background:**

Alpha-1-Antitrypsin (A1AT) is a protease inhibitor encoded by the SERPINA1 gene. A1AT serves as the primary natural inhibitor of Proteinase 3 (PR3), an enzyme found in neutrophils. PR3 is an antigenic target of Anti-Neutrophil Cytoplasmic Antibodies (ANCA). While numerous studies have established a connection between Single Nucleotide Polymorphisms (SNPs) in the SERPINA1 gene and ANCA-Associated Vasculitis (AAV), limited research has delved into the impact of these polymorphisms on the prognosis of these patients.

**Objective:**

The present study's objective is to investigate mortality disparities among Brazilian AAV patients carrying SERPINA1 SNPs (rs7151526, rs28929454) compared to non-carriers. Additionally, the authors analyzed demographic, clinical, and serologic data in these two groups.

**Methods:**

In this single-center prospective cohort study, the authors enrolled AAV patients who were monitored for a duration of up to three years. The identification of SNPs was conducted through RT-PCR. Survival analysis, including Kaplan-Meier survival curves, and Cox proportional regression analysis was subsequently performed to evaluate outcomes.

**Results:**

The authors assessed 115 patients (65.2% with granulomatosis with polyangiitis, 17.4% with eosinophilic granulomatosis with polyangiitis, and 17.4% with microscopic polyangiitis). All patients were aged ≥ 18 years, with 37.4% being female, and 54.7% identified as White. The association between SERPINA1 SNPs proved to be the most significant factor linked to mortality in the cohort (HR = 6.2, 95% CI 1.4‒27.1, p = 0.015). SERPINA1 SNP carriers exhibited a lower mean survival [rs7151526: 57.4 (42.7‒72.2) years, p < 0.007; rs28929454: 54.9 (40.9‒68.9) years, p < 0.0001] than non-carriers (68.0 [67.2‒69.0] years).

**Conclusion:**

SERPINA1 SNPs are associated with increased mortality in Brazilian AAV patients.

## Highlights

• The SERPINA1 gene encodes Alpha-1-Antitrypsin (A1AT), a protease inhibitor.
• SERPINA1 gene polymorphisms are associated with ANCA-associated vasculitis.
• Their presence represents poor prognostic factors in ANCA-associated vasculitis.

## Introduction

Antineutrophil Cytoplasmic Antibody (ANCA)-Associated Vasculitis (AAV) is a group of vasculitis that predominantly affects small vessels and encompasses Granulomatosis with Polyangiitis (GPA), Microscopic Polyangiitis (MPA) and Eosinophilic Granulomatosis with Polyangiitis (EGPA). GPA is more common in populations of Caucasian origin, whereas MPA is more common in populations of Asian origin.^1^, ^2^, ^3^

Despite their similarities, these three entities do not represent different manifestations of the same disease. Each presents clinical, laboratory, and histological characteristics that distinguish them.^4^ Up to 90 % of patients with generalized GPA or MPA are positive for ANCA, with Proteinase 3 (PR3)-ANCA being more associated with GPA and Myeloperoxidase (MPO)-ANCA with MPA.^5^ ANCAs are directly related to the pathophysiogenesis of these diseases.^4^^,^^6^

The three AAVs are polygenic diseases. The Human Leukocyte Antigen (HLA) loci are the most associated with the AAVs, especially HLA-DPA1 and DPB1 with GPA/PR3-ANCA, and HLA-DQA2 and DQB1 with MPA/MPO-ANCA.^3^^,^^7^, ^8^, ^9^, ^10^, ^11^ In the Japanese population, HLA-DRB1 is associated with MPA/MPO-ANCA.^12^ Regarding EGPA, studies have shown an association between AAV and HLA-DRB4, DRB1, and DQ.^13^, ^14^, ^15^, ^16^

PR3 is encoded by the PRTN3 gene located on chromosome 19, and its main natural inhibitor is Alpha-1-Antitrypsin (A1AT), a protease inhibitor encoded by the SERPINA1 gene located on chromosome 14.^7^^,^^11^ The presence of the Z-allele (single nucleotide polymorphism, SNP, rs28929474) of SERPINA1 in homozygosity leads to Alpha-1-Antitrypsin Deficiency (A1ATD).^17^ Studies have also demonstrated a higher frequency of Z-allele carriers in Caucasian GPA cohorts.^18^, ^19^, ^20^, ^21^ More recently, two Genome-Wide Association Studies (GWAS) confirmed the association between SERPINA1 SNPs with AAV, especially GPA and anti-PR3 antibody positivity.^8^^,^^9^ However, large studies including non-Caucasian or mixed populations are lacking.

The Z-allele is present in 4 % of individuals of European descent.^17^ The frequency of this allele and prevalence of A1ATD in the multiethnic Brazilian population remain unknown.

While several studies have identified an association between SERPINA1 gene SNPs and ANCA-Associated Vasculitis (AAV),^18^, ^19^, ^20^, ^21^ there is a scarcity of data regarding the influence of these SNPs on the prognosis of affected individuals. The objective is to examine mortality differences among Brazilian AAV patients carrying SERPINA1 SNPs in comparison to non-carriers. Furthermore, the authors conducted a comprehensive analysis of demographic, clinical, and serological data of these two groups.

## Material and methods

A single-center prospective cohort study was conducted, including 115 patients followed up in the tertiary center, during the study period (June 2018 to June 2021), with AAV diagnosis made by a rheumatologist or pneumologist, based on clinical manifestations, laboratory data, histopathological and imaging findings. Patients with (AAV) were followed for up to three years after the enrollment of the first patient in the study (from June 2018 to June 2021).

Inclusion criteria: The authors included patients with AAV who were Black or White, ≥18 years old, and who agreed to participate in the study after signing an Informed Consent Form. Exclusion criteria: Patients aged <18 years old and of other ethnicities were excluded from the study.

Death due to disease activity, infection, cardiovascular disease, neoplasia, or an unknown cause was the primary outcome evaluated. The SERPINA1 gene SNPs investigated were chosen according to the two main GWAS^8^^,^^9^ available in the literature: SERPINA1 rs7151526 (NC_000014.9:g.94397299C>A) and rs28929474 (NC_000014.9:g.94378610C>G).

Patients were classified as having GPA, MPA, or EGPA according to the 2012 revised International Chapel Hill Consensus Conference Nomenclature of Vasculitides^22^ and the European Medicines Agency's Algorithm for Classification of ANCA-Associated Vasculitis.^23^

Sex was categorized as male or female according to birth designation. To define ethnicity, patients were asked about the skin color of their paternal and maternal grandparents.^24^^,^^25^ Patients who reported that they were White were classified as White. Those who had at least one Black grandfather were classified as Black. For those who did not know the colors of their grandparents, the authors only considered their parents’ colors.

To quantify disease activity and damage, the Birmingham Vasculitis Activity Score (BVAS)^26^ and Vasculitis Damage Index (VDI)^27^ clinical scores were used, respectively. The included patients underwent routine follow-up, and the frequency of consultations was determined by the attending physician based on the severity of each case.

Peripheral blood samples were obtained from each patient at the time of enrollment. The DNA was extracted from whole blood samples using a Qiagen kit (QIAGEN GmbH, Hilden, Germany), and the processed samples were subsequently stored at −20 °C. SNP genotyping was conducted utilizing the TaqMan SNP Genotyping Assays (Applied Biosystems, Foster City, CA) and the StepOne Plus equipment (Applied Biosystems, Foster City, CA). For the SNP rs28929474, the authors used the Assay ID C_34508510_10, and for the SNP rs7151526, we used the Assay ID C_29386835_10. The authors rigorously followed the manufacturer's protocol.

The presence of ANCAs was assessed using indirect immunofluorescence (perinuclear vs. cytoplasmic) and ELISA (anti-MPO and anti-PR3 antibodies, Inova Diagnostics, Werfen, Brazil). A1AT (mg/dL) levels were determined using an immunoturbidimetric method.

The study was approved by the local Ethics Committee (CAAE: 18393119.7.0000.0068) and has been carried out in accordance with The Code of Ethics of the World Medical Association (Declaration of Helsinki) for experiments involving humans.

### Statistical analyses

Continuous variables were tested for normality using the Kolmogorov-Smirnov and Shapiro-Wilk tests and were determined to be non-parametric. Continuous and semi-continuous values were expressed as medians (25^th^–75^th^). Categorical data were presented as absolute values and percentages and were analyzed using Pearson's Chi-Square test or Fisher's exact test when necessary. Non-parametric data were compared using the Mann-Whitney *U* test for two independent samples or the Kruskal-Wallis test with a Müller-Dunn post-hoc test for three or more samples.

The discrimination of continuous and semicontinuous variables was performed using the ROC Curve (Operational Response Curve) through the area under the curve and asymptotic significance. Continuous variables without an established cut-off value endorsed by three or more international articles were categorized using the ROC curve. The cut-off points and score for each variable were determined by the highest value of the sum of the sensitivity and specificity, which corresponded to the point of greatest inflection of the ROC curve.

Kaplan-Meier Survival analysis and survival curves were differentiated using the log-rank test, and life expectancies were expressed as the mean and standard error of the estimate.

Cox proportional regression analysis was performed considering variables that had a significance value of < 0.2 by the log-rank test in the Kaplan-Meier survival analysis. Predictive variables were those with a Hazard Ratio (HR) value (whose lower and upper values of confidence intervals did not exceed one) that were significant (*p* < 0.05).

The authors considered risk α < 0.05 for committing a type I or 1st type error, and risk β < 0.20 for committing a type II or 2nd type error.

Statistical analyses were performed using SPSS 21.0, IBM®, and GraphPad Prism 9.0 GraphPad®.

## Results

This study included 115 patients, 75 of whom were classified as having GPA (65.2 %), 20 (17.4 %) as having MPA, and 20 (17.4 %) as having EGPA. All patients were aged ≥18 years, 37.4 % were female, and 54.7 % were White. The demographic, clinical, and immunological characteristics of the patients are shown in Table 1.

**Table 1:** Descriptive data of the cohort (115 patients).

Demographic data		n (%)
Ethnicity	White	63 (54.7)
	Black	52 (45.3)
Age (decades)	18‒29	8 (7.0)
	30‒39	16 (13.9)
	40‒49	22 (19.1)
	50‒59	26 (22.6)
	60‒69	28 (24.3)
	70‒85	15 (13.0)
Sex	Women	43 (37.4)
	Men	72 (62.6)
**Disease classification**		**n (%)**
Granulomatosis with polyangiitis		75 (65.2)
Eosinophilic granulomatosis with polyangiitis		20 (17.4)
Microscopic polyangiitis		20 (17.4)
**Clinical manifestations**		**n (%)**
Central nervous system^a^		9 (7.8)
Peripheral nervous system^b^		36 (31.3)
Ocular manifestations^c^		24 (20.9)
Rhinossinusitis		82 (71.3)
Lung nodules		41 (35.7)
Alveolar hemorraghe		22 (19.1)
Interstitial lung disease		4 (3.5)
Bronchiectasias		13 (11.3)
Glomerulonephritis		56 (48.7)
Arthritis		22 (19.1)
Cutaneous lesions^d^		37 (32.2)
Asthma		20 (17.4)
**Comorbidities**		**n (%)**
Hypertension		59 (5.3)
Glomerular filtration rate < 50 mL/min		21 (18.3)
End-stage renal disease^e^		15 (13.0)
Diabetes		15 (13.0)
**Death**		**n (%)**
Death		11 (10.7)
Cause of death	Disease activity	4 (36.4)
	Infection	5 (45.5)
	CVD^f^	1 (9.0)
	Malignancy	0
	Unknown	1 (9.0)
**ANCA**^g^		**n (%)**
Positive anti-myeloperoxidase		20 (17.4)
Positive anti-proteinase 3		39 (33.9)
ANCA indirect immunofluorescence	Negative	19 (16.5)
	Cytoplasmic	65 (56.5)
	Perinuclear	31 (27.0)
**Genetic profile (SNP**^h^**)**		**n (%)**
SERPINA1 rs7151526 (A)^i^	CC	106 (92.2)
	CA	7 (6.1)
	AA	2 (1.7)
SERPINA1 rs28929474 (A)	CC	106 (92.2)
	CA	7 (6.1)
	AA	2 (1.7)
**Continuous variables**		**Median (25 %**‒**75 %)**
*Vasculitis damage index*		4 (2‒6)
Maximum *Birmingham vasculitis activity index*^j^		2 (0‒7)
Age (years)		53 (43‒63)
Follow-up (years)^k^		5 (2‒12)

a Vasculitis, aseptic meningitis, pachymeningitis.

b Distal symmetric polyneuropathy, mononeuritis multiplex.

c Uveitis, episcleritis, escleritis, inflammatory orbital pseudotumor.

d Purpura, ulcers, nodules.

e Creatinine clearance < 15 mL/min or on hemodialysis.

f Cardiovascular disease.

g Anti-neutrophil cytoplasmic antibodies.

h SNP, Single Nucleotide Polymorphism.

i The letter A represents the alleles with the SNPs; the letter T represents the alleles without the SNPs.

j Higher Birmingham vasculitis activity index value in the two years prior to inclusion in the study.

k Considering the period from diagnosis to inclusion in the study plus the period in which the patient was followed up in the study.

The rs7151526 and rs28929474 (Z-allele) were not in Hardy-Weinberg Equilibrium (HWE) in the present cohort. They were in linkage disequilibrium with an association coefficient of 0.88 (*p* < 0.001).

### Allelic analysis: demographic data, disease classification, clinical manifestations, and ANCA pattern

SERPINA1 SNP rs28929474: 27.3 % of the carriers of this SNP were female (*p* = 0.477), 72.7 % were black (*p* = 0.060), and 72.7 % were younger than 50 years (*p* < 0.001). Regarding the diagnosis, 72.7 % of the carriers were classified as having GPA (*p* = 0.592), and 27.3 % as MPA (*p* = 0.376). There were no cases of EGPA among the carriers of this SNP (Table 2). The authors did not observe statistically significant differences between the groups in terms of clinical manifestations and ANCA patterns (Table 3).

**Table 2:** Allelic analysis according to age, ethnicity, sex and classification of the disease.

		SERPINA1 SNP^a^ (rs7151526)				SERPINA1 SNP (rs28929474)						Rs71	Rs28
		C^b^ (71)		A^b^ (71)		C (28)		A (28)		Total		χ^2^	χ^2^
		n	%	n	%	n	%	n	%	n	%	p-value	p-value
**Allelic distribution (2n)**		219	95	11	5	219	95	11	5	230	100		
**Age (decades of life)**	18‒29	10	4.6	6	54.5	10	4.6	6	54.5	16	7.0	0.001	0.001
	30‒39	32	14.6	0	0.0	32	14.6	0	0.0	32	13.9		
	40‒49	41	18.7	3	27.3	42	19.2	2	18.2	44	19.1		
	50‒59	51	23.3	1	9.1	51	23.3	1	9.1	52	22.6		
	60‒69	56	25.6	0	0.0	55	25.1	1	9.1	56	24.3		
	70‒85	29	13.2	1	9.1	29	13.2	1	9.1	30	13.0		
**Ethnicity**	White	124	56.6	2	18.2	123	56.2	3	27.3	126	54.8	0,012	0,060
	Black	95	43.4	9	81.8	96	43.8	8	72.7	104	45.2		
**Sex**	Female	83	37.9	3	27.3	83	37.9	3	27.3	86	37.4	0.477	0.477
	Male	136	62.1	8	72.7	136	62.1	8	72.7	144	62.6		
**Classification**	GPA^c^	142	64.8	8	72.7	142	64.8	8	72.7	150	65.2	0.592	0.592
	EGPA^d^	40	18.3	0	0.0	40	18,3	0	0.0	40	17.4	0.119	0.119
	MPA^e^	37	16.9	3	27.3	37	16,9	3	27.3	40	17.4	0.376	0.376

a Single nucleotide polymorphism.

b Letter C represents the allele without the SNPs and letter A represents the alleles with the SNPs.

c Granulomatosis with polyangiitis.

d Eosinophilic granulomatosis with polyangiitis.

e Microscopic polyangiitis. Statistically significant: p-value < 0.05.

**Table 3:** Allelic analysis according to clinical manifestations and ANCA pattern.

		SERPINA1 SNP^a^ rs7151526 (A)			SERPINA1 SNP^a^ rs28929474 (A)	
	**C**^b^	**A**^b^		**C**^b^	**A**^b^	
	**n (%)**	**n (%)**	**p-value**	**n (%)**	**n (%)**	**p-value**
**Allelic distribution (2n)**	**219 (95 %)**	**11 (5 %)**		**219 (95 %)**	**11 (5 %)**	
**Clinical manifestations**						
Rhinosinusitis	156 (71.2)	8 (72.7)	0.915	156 (71.2)	8 (72.7)	0.915
Lung nodule	77 (35.2)	5 (45.5)	0.487	77 (35.2)	5 (45.5)	0.487
Alveolar hemorrhage	40 (18.3)	4 (36.4)	0.136	40 (18.3)	4 (36.4)	0.136
Glomerulonephritis	107 (48.9)	5 (45.5)	0.826	106 (48.4)	6 (54.5)	0.691
**ANCA**^c^**pattern**						
MPO-ANCA^d^ +	37 (16.9)	3 (27.3)	0.376	37 (16.9)	3 (27.3)	0.376
PR3-ANCA^e^ +	75 (34.2)	3 (27.3)	0.634	75 (34.2)	3 (27.3)	0.634
Negative ANCA	38 (17.4)	0	0.302	38 (17.4)	0	0.302
c-ANCA^f^	123 (56.2)	7 (63.6)	0.302	123 (56.2)	7 (63.6)	0.302
p-ANCA^g^	58 (26.5)	4 (36.4)	0.302	58 (26.5)	4 (36.4)	0.302

a Single nucleotide polymorphism.

b Letter C represents the allele without the SNPs and letter A represents the alleles with the SNPs.

c Anti-neutrophil cytoplasmic antibodies.

d Anti-myeloperoxidase ANCA.

e Anti-proteinase-3 ANCA.

f Cytoplasmic pattern ANCA.

g Perinuclear pattern ANCA. Statistically significant: p-value < 0.05.

SERPINA1 SNP rs7151526: 27.3 % of carriers were female (*p* = 0.477), 81.8 % were black (*p* = 0.012), and 81.8 % were younger than 50 years (*p* < 0.001). Regarding diagnosis, 72.7 % of the carriers were classified as having GPA (*p* = 0.592), and 27.3 % as having MPA (*p* = 0.376). There were no cases of EGPA among carriers of this SNP (Table 2). The authors did not observe statistically significant differences between the groups in terms of clinical manifestations and ANCA patterns (Table 3).

### Serum levels of A1AT

Serum levels of A1AT were significantly lower and below the normal range (90‒200 mg/dL) in patients homozygous for rs28929474 than in non-carriers [24.5 (20‒29) mg/dL vs. 143 (128.5‒163.5) mg/dL, p = 0.010]. Carriers of the same SNP in heterozygosis also had lower serum levels than non-carriers but with the median still within the normal range [90 (68‒130) mg/dL vs. 143 (128.5‒163.5) mg/dL, *p* = 0.007]. The data regarding rs7152526 were very similar because of the linkage disequilibrium between these SNPs (not shown).

### Survival analysis

Considering the time interval between diagnosis and enrollment in the study, along with the study time interval, the patients were followed up for a median of 5 (2‒12) years. Eleven patients died during the study period, with infection being the leading cause of death (45 %), followed by disease activity (36 %), cardiovascular disease (9 %), and unknown causes (9 %) (Table 1).

Univariate analysis of the comparison of demographic, clinical, and immunological characteristics between survivors and non-survivors is presented in Table 4. Diabetes mellitus (*p* = 0.002), end-stage renal disease (*p* = 0.030), bronchiectasis (*p* = 0.017), ocular involvement (*p* = 0.011), and ANCA-positivity (*p* = 0.026) were associated with mortality.

**Table 4:** Univariate analysis: survivors versus non-survivors’ patients.

Variables	Survivors		Non-survivors		p-value
	n	%	n	%	Pearson χ^2^
Men	122	61.0	18	75.0	0.181
Diabetes mellitus	22	11.0	8	33.3	0.002
End stage renal disease^a^	20	10.0	6	25.0	0.030
Bronchiectasis	18	9.0	6	25.0	0.017
Lung nodule	34	17.0	8	33.3	0.053
Rhinossinusistis	148	74.0	14	58.3	0.105
Ocular manifestations	38	19.0	10	41.7	0.011
Positive ANCA^b^	156	81.3	22	100.0	0.026
	**Median**	**25th**‒**75 ^th^**	**Median**	**25 ^th^**‒**75 ^th^**	**Mann-Whitney**
Age (years)	52.0	41.0‒62.0	61.5	49.5‒66.0	0.019
Maximum BVAS^c^ (2 years)^d^	1.0	0.0‒6.0	4.0	0.0‒10.5	0.139

a Creatinine clearance < 15 mL/min or on hemodialysis.

b Anti-neutrophil cytoplasmic antibodies.

c Birmingham Vasculitis Activity Score.

d Highest BVAS value reached in the last 2 years before inclusion in the study. Statistically significant: p-value < 0.05.

**Fig. 1:**
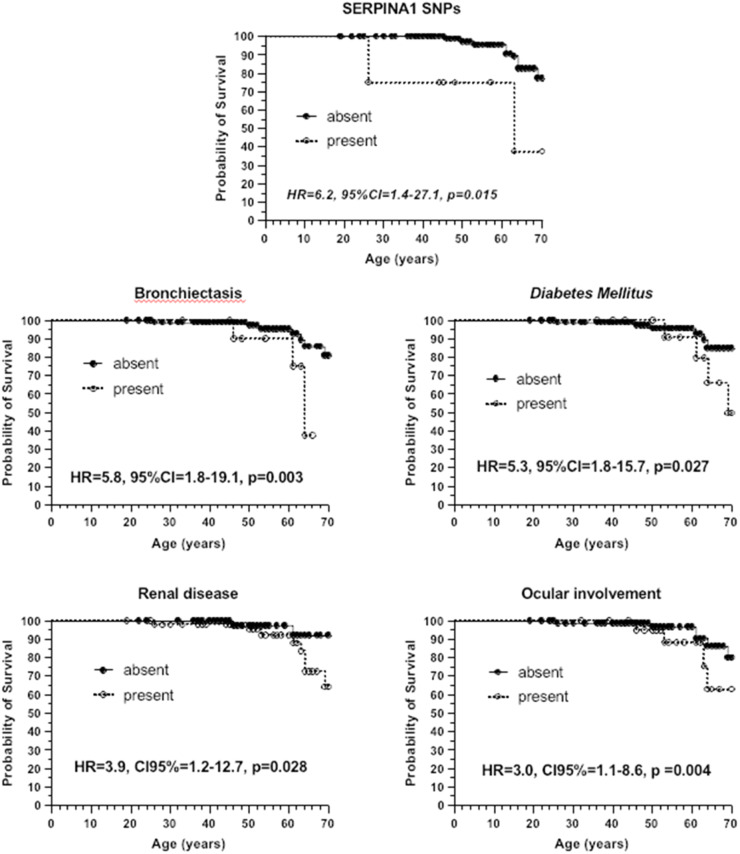
Cox proportional hazard model: genetic factor and comorbidities. HR, Hazard Ratio; CI, Confidence Interval. The analysis of the association of the SNPs of the SERPINA1 gene was performed due to the linkage disequilibrium between them (association coefficient 0.88, *p* < 0.001). Statistically significant: p-value < 0.05.

In the Cox proportional hazards model (Fig. 1), the SERPINA1 SNPs association was the most strongly factor associated with mortality (HR = 6.2, 95 % CI 1.4‒27.1, *p* = 0.015), followed by bronchiectasis (HR = 5.8, 95 % CI 1.8‒19.1, *p* = 0.003), diabetes mellitus (HR = 5.3, 95 % CI 1.8‒15.7, *p* = 0.027), end-stage renal disease (HR = 3.9, 95 % CI 1.2‒12.7, *p* = 0.028), and ocular involvement (HR = 3.0, 95 % CI 1.1‒8.6, *p* = 0.004).

Comparative analysis of survival between SERPINA1 SNPs carriers and non-carriers showed that carriers of both SNPs had a lower mean survival [rs7151526: 57.4 (42.7‒72.2) years, *p* < 0.007; rs28929454: 54.9 (40.9‒68.9) years, *p* < 0.0001] than non-carriers [68.0 (67.2‒69.0) years] (Table 5).

**Table 5:** Comparative analysis of survival between SERPINA1 SNP carriers vs. non-carriers’ patients.

Kaplan-Meier	Mean	Standard error	95 % CI lower limit	95 % CI upper limit	Log-rank (Mantel-Cox)
General	67.7	0.5	66.7	68.7	
SERPINA1 rs7151526					
C^a^	68.0	0.4	67.2	68.9	*p* < 0.007
A^a^	57.4	7.5	42.7	72.2	
SERPINA1 rs28929474					
C	68.1	0.4	67.2	69.0	*p* < 0.0001
A	54.9	7.1	40.9	68.9	

a Letter C represents the alleles without the SNP and letter A represents the alleles with the SNP. Statistically significant: p-value < 0.05.

Regarding disease classification, no differences in survival were found between the groups. GPA patients had a mean survival of 67.6 (66.2‒69.0) years; MPA patients 66.6 (67.7‒70.3) years; and EGPA patients 69.0 (67.7‒70.3) years (GPA vs. MPA *p* = 0.508; GPA vs. EGPA *p* = 0.236; MPA vs. EGPA *p* = 0.129).

When the authors considered the ANCA pattern, no differences in survival were found between PR3/c-ANCA patients [67.4 (65.9‒68.9) years] vs. MPO/p-ANCA patients [67.2 (65.4‒69.0) years, *p* = 0.793]. However, ANCA negative patients had slightly higher survival when compared to ANCA positive patients [ANCA negative: 69.3 (68.1‒69.4) years vs. MPO/p-ANCA positive 67.2 (65.4‒69) years, *p* = 0.036; vs. PR3/c-ANCA positive 67.4 (65.9‒68.9) years, *p* = 0.043].

Regarding ethnicity, no differences in survival were found between the groups [White patients had a mean survival of 66.4 (67.5–69.3) years, and Black patients 66.4 (64.1‒68.7) years, *p* = 0.291].

## Discussion

This is the first study to evaluate the association between SERPINA1 SNPs and mortality in a Brazilian multiethnic cohort of patients with AAV. In addition to examining demographic, clinical, and immunological parameters, the present research expands beyond the scope of prior studies, which primarily focused on populations comprised predominantly of White patients.^8^^,^^9^^,^^18^, ^19^, ^20^, ^21^ The results of the present study confirm the association of SERPINA1 SNPs, especially the Z-allele, with higher mortality in Brazilian patients.

SERPINA1 SNPs were not found in HWE in this cohort. This is probably due to their association with higher mortality, as the authors demonstrated in the survival analysis.

The present study also showed that these SNPs did not have independent segregation in the Brazilian population, as there was an association of > 80 % between them. Lyons et al.^9^ also demonstrated linkage disequilibrium between these SNPs in a White population. In our opinion, the Z-allele is most likely the variant that is important in the pathophysiogenesis of AAV. A1AT deficiency is one of the factors responsible for the increased availability of PR3, facilitating its processing and presentation via HLA class II to T helper lymphocytes, with the consequent breakdown of immunological tolerance.

When comparing patients who were carriers of both SERPINA1 SNPs with non-carriers, the authors found that their frequencies were higher in those diagnosed at < 50 years of age and in Black patients. In this sample, two individuals who were homozygous for both polymorphisms were aged < 30 years and Black. These data highlight the need to consider the diagnosis of A1ATD in young patients with AAV, regardless of ethnicity.

Compared to non-carriers, patients heterozygous for both SERPINA1 polymorphisms had significantly lower serum A1AT concentrations. However, the median concentrations were within the normal ranges. Only patients who were homozygous for both polymorphisms had consistently lower serum concentrations. These results are similar to those reported by Deshayes et al.,^28^ who compared patients with AAV carriers with non-carriers of the Z-allele. The authors reinforced that the Z-allele is responsible for the deficient production of A1AT, and the findings regarding rs7151526 are due to linkage disequilibrium between these two SNPs.

Serum levels of A1AT were shown to be increased in patients with active AAV, both in anti-PR3- and anti-MPO-positive individuals.^29^ A1AT is an acute-phase protein similar to C-reactive protein, that increases inflammatory processes. The authors conclude that, despite not being able to properly identify heterozygous patients, A1AT measurement can be used as a screening method for A1ATD in patients with AAV to identify possible homozygotes for the Z-allele, especially in young patients.

Patients with AAV have a higher risk of death than the general population. A recent meta-analysis^30^ demonstrated a 2.7 times greater risk, with an emphasis on deaths from infection, especially in the first few years of the disease when immunosuppressive treatment is more aggressive, and cardiovascular diseases.^31^ In the cohort, the main cause of death was infection followed by disease activity.

Advanced age, severe cardiac, renal, and gastrointestinal involvement, higher damage and activity scores, and renal biopsy with sclerotic patterns are examples of mortality predictors described in the literature.^32^, ^33^, ^34^ A recent retrospective study by the group also demonstrated that renal dysfunction was a predictor of mortality in the population.^35^

Higher mortality among patients with AAV carrying the Z-allele has been demonstrated in two retrospective studies by Elzouki et al.^19^ and Segelmark et al.^36^ The present study prospectively evaluated patients with AAV and identified an important association between SERPINA1 SNPs and lower life expectancy. The data are virtually identical for the two SNPs because of the linkage disequilibrium between them. In the Cox proportional hazards model, these SNPs were the main factors associated with mortality in the cohort.

In the cohort, diabetes mellitus and end-stage renal disease, which are recognized risk factors for mortality in the general population,^37^^,^^38^ were also associated with higher mortality in patients with AAV. In addition, bronchiectasis and ocular involvement were associated with higher mortality rates. Choi et al. demonstrated an increased risk of death in patients with bronchiectasis and other comorbidities (obstructive pulmonary disease, lung cancer, and cardiovascular disease).^39^ One hypothesis that may justify this finding is that bronchiectasis increases the risk of pulmonary infectious complications in highly immunosuppressed patients.

It was not possible to establish an association between the presence of the studied SNPs, AAV classification (GPA vs. MPA vs. EGPA), and ANCA specificity. In our service, for many years, the authors only had ANCA measurements available by indirect immunofluorescence and, more recently, measurement by the ELISA method was introduced. It is possible that many patients had negative ELISA results because they had already received immunosuppressive treatments.

An important limitation of the present study is the difficulty in establishing ethnicities in a country as mixed as Brazil. It is likely that many White patients have Black descendants or even descendants of other ethnicities. In addition, to simplify the analysis, the authors used only the classification of White and Black, and most patients included in both categories could be classified as mixed race. Another limitation is that the authors did not use the Sanger method for the identification of the SNPs. However, the identification of SERPINA1 SNPs by TaqMan SNP Genotyping is a well-validated method that has been used in other important studies.^40^

## Conclusion

This study is the first to demonstrate an association between SERPINA1 gene SNPs (rs7151526 and rs28929474) and higher mortality in a Brazilian multiethnic cohort of AAV patients. The presence of these SNPs was also more frequent in younger individuals (< 50 years) and Black patients.

## Data availability

The data that support the findings of this study are available on request from the corresponding author, [initials].

## CRediT authorship contribution statement

**Henrique Ayres Mayrink Giardini:** Conceptualization, Methodology, Data curation, Investigation, Writing – original draft. **Valeria de Falco Caparbo:** Conceptualization, Data curation, Investigation. **Isac de Castro:** Conceptualization, Methodology, Formal analysis, Writing – review & editing. **Andréia Padilha Toledo:** Investigation, Data curation. **Carmen Silvia Valente Barbas:** Conceptualization, Methodology. **Samuel Katsuyuki Shinjo:** Supervision, Writing – review & editing. **Rosa Maria Rodrigues Pereira:** Conceptualization, Methodology, Supervision, Project administration, Writing – review & editing.

## Declaration of competing interest

The authors declare no conflicts of interest.
